# COVID-19 Vaccine Hesitancy in Poland—Multifactorial Impact Trajectories

**DOI:** 10.3390/vaccines9080876

**Published:** 2021-08-07

**Authors:** Paweł Sowa, Łukasz Kiszkiel, Piotr Paweł Laskowski, Maciej Alimowski, Łukasz Szczerbiński, Marlena Paniczko, Anna Moniuszko-Malinowska, Karol Kamiński

**Affiliations:** 1 Department of Population Medicine and Lifestyle Diseases Prevention, Medical University of Bialystok, Waszyngtona 13A, 15-089 Białystok, Poland; sowa@umb.edu.pl (P.S.); m.paniczko@gmail.com (M.P.); 2 Society and Cognition Unit, University of Bialystok, 15-403 Bialystok, Poland; lukaszkiszkiel@gmail.com (Ł.K.); pio.laskowski@gmail.com (P.P.L.); 3 Doctoral School of Social Sciences, University of Bialystok, 15-403 Bialystok, Poland; m.alimowski@uwb.edu.pl; 4 Department of Endocrinology, Diabetology and Internal Medicine, Medical University of Bialystok, 15-276 Bialystok, Poland; lukasz.szczerbinski@umb.edu.pl; 5 Clinical Research Centre, Medical University of Bialystok, 15-276 Białystok, Poland; 6 Department of Infectious Diseases and Neuroinfections, Medical University of Bialystok, 15-089 Białystok, Poland; annamoniuszko@op.pl; 7 Department of Cardiology, University Hospital of Bialystok, 15-276 Białystok, Poland

**Keywords:** vaccine hesitancy, COVID-19, vaccine acceptability, SARS-CoV-2

## Abstract

Since the declaration of the SARS-CoV-2 pandemic confirmed by World Health Organization, work on the development of vaccines has been stimulated. When vaccines are commonly available, a major problem is persistent vaccine hesitancy in many European countries. The main goal of our study was to understand the multidimensional factors inducing this phenomenon in Poland. Our study was carried out at the third wave’s peak of the pandemic, with record rates of daily cases and deaths associated with COVID-19. The results indicate that vaccine hesitancy/acceptability should always be considered in an interdisciplinary manner and according to identified factors where most negative attitudes could be altered. Our analyses included the assessment of a representative quota sample of adult Poles (*N* = 1000). The vaccine hesitancy in the studied group reached 49.2%. We performed stepwise logistic regression modeling to analyze variables set into six trajectories (groups) predicting the willingness to vaccinate. Apart from typical, socio-demographic and economic determinants, we identified the fear of vaccines’ side effects, beliefs in conspiracy theories and physical fitness. We were also able to establish the order of importance of factors used in a full model of all impact trajectories.

## 1. Introduction

The pandemic of COVID-19 was declared by WHO in March 2020 and, as a consequence, restriction policies (“lockdowns”) were established in majority of countries. It has undoubtedly been affecting societies in all possible dimensions: social, mental, health, economic, professional and family. The exact effect of the pandemic on human population will be studied for many years. Due to the interdisciplinarity of the pandemic’s affectivity, while understanding changes in human health behavior, a multidimensional analysis must be assumed. Such an approach also applies to the search for causal links to attitudes towards vaccination against COVID-19.

Since the incidence of SARS-CoV-2 has become a cross-border issue, pharmaceutical companies and scientists began an intensive collaboration to produce an effective vaccine. Currently, several of these vaccines have already been approved for use in parallel with the ongoing third phase of clinical trials [1,2]. In the last year, there has been a substantial interest of scientists in finding the most adequate methods of ending the pandemic. It seems that this emerging issue focuses on vaccinating as much of the population as possible and relying on those immunized after recovery from infection in hope that these measures will reduce the risk of virus transmission [3,4]. However, the current problem is COVID-19 vaccine hesitancy, which is being reported in an increasing number of scientific papers.

The World Health Organization defines vaccine hesitancy as “delay in acceptance or refusal of safe vaccines despite availability of vaccine services” [5,6]. This issue has been perceived in various contexts for many years regarding the impact on population health, its determinants, as well as possible strategies to oppose them [7]. A well-known example is the so-called 5C model of the drivers of vaccine hesitancy assessing individual person-level determinants: confidence, complacency, convenience, risk calculation and collective responsibility. This model has been introduced as a framework for psychological antecedents of vaccination in high-income countries, but it has not been validated and implemented in Polish research so far [8,9]. The main goal of our study was to understand the multidimensional factors inducing vaccine hesitancy or acceptability in Poland, belonging to the group of Central and Eastern European countries with the lowest vaccination acceptability [10,11]. Our study was carried out at the third wave’s peak of the pandemic, with record rates of daily cases and deaths associated with COVID-19 [12]. Our results indicate that vaccine hesitancy/acceptability should always be considered in an interdisciplinary manner, and according to identified factors, the majority of negative attitudes could be modified.

## 2. Materials and Methods

The primary source of data for this paper was the project ‘Rise or fall? Short and long-term health and psychosocial trajectories of COVID-19 pandemics’ granted by the Polish National Science Centre. In this interdisciplinary project, the impact of COVID-19 on the general population is going to be measured over 4 years. Our analyses included a part of that data with the assessment of quota sample of adult Poles (*N* = 1000). 

We used an online Computer-Assisted Web-Interviewing (CAWI) questionnaire to collect data between 12 and 23 of March. It was extracted and processed in early April 2021, i.e., during the third COVID wave in Poland and at the beginning of the nationwide vaccination campaign. Furthermore, this was exactly a year since the first COVID-19 cases had been detected in Poland. At that time, the CAWI technique was the only one that could be used to carry out a 35-min survey on a nationwide sample of Poles. The questionnaire consisted of 141 closed questions arranged in 21 thematic blocks concerning the individual areas of the respondents’ functioning before and during the pandemic to capture a possible change and its direction. The CAWI script fully controlled the flow of the survey and its filters, which stopped the respondents from missing the questions; as a result, there were no missing data.

The questionnaire was composed of the following sections: metrics; subjective assessment of health and fitness before and during the pandemic; coronavirus testing and quarantine; in-depth health interview; perceived pain; nutrition; social life; religion; evaluation of the activities of institutions and government; social distance, hygiene and disinfection; vaccinations; work and employment; interest in events and stress; alcohol and cigarettes; patients with COVID-19 (only those who indicated that they had been suffering from the disease); children and family; beliefs and attitudes towards the COVID-19 pandemic—including the conspiracy theories; the financial situation of the household; health competences; summary of the impact of the pandemic on the functioning of the individual. The survey was created based on the following international surveys: European Social Survey (https://www.europeansocialsurvey.org/ accesed 29 July 2021); The Joint Research Center COVID-19 Survey (European Comission’s science and knowledge center—https://ec.europa.eu/eusurvey/runner/JRC-Covid19-Survey accesed 29 July 2021); and International Social Survey Program (ISSP—Health module, http://www.issp.org/menu-top/home/ accesed 29 July 2021). Additionally, the survey used the indicators from the ‘Bialystok Plus’ longitudinal cohort study conducted at the Medical University of Bialystok (https://bialystok.plus/en/english/ accesed 29 Julty 2021).

The sample was balanced in terms of national quotas for age, gender, voivodships (regions), class of residence and education level. The respondents were drawn from the largest opinion panel website in Poland (opinie.pl with over 125,000 active panelists), whose provider IQS Group is affiliated to OFBOR (Polish Association of Public Opinion and Marketing Research Firms) and has ISO and PKJPA (Interviewer Work Quality Control Program) certificates in the CAWI technique. The drawing was carried out until the quotas were filled, after which the results were adjusted to match the structure of the general population of Poles.

### 2.1. Design and Sample Selection 

All demographic data used to create the sample structure came from the Central Statistical Office. They are available at the Local Data Bank (BDL) https://bdl.stat.gov.pl/BDL/start, accessed on 29 July 2021. The demographic database of the agency carrying out the survey was built based on the most recent data at that time (from December 2020). The database was a combination of several databases published in the BDL: 1. Population by individual age and sex-data at the commune level; 2. Population by age and sex groups-data at the commune level; 3. Population by individual age and sex (semi-annual data)—data at the voivodship level; 4. Percentage of the population aged 15 and more by education level, sex and place of residence—data at the voivodship level.

Based on the above-mentioned data sources, a demographic database was built containing data of the population of Poland at the level of communes—the lowest available aggregation level—intersected by the typical demographic variables—gender, age, voivodship, size of the place of residence and level of education.

The sample for the study was created based on the quotas for the defined variables, i.e., gender and age, which were intersected, and the border distributions for voivodships, size of the place of residence and level of education. The distribution of characteristics in the population obtained in the sample generation process was used to control the sample of the survey carried out on the panel and to weigh the results. Respondents were invited to complete the questionnaire by the research agency via e-mail with a link to the study. In total, 1267 invitations were sent out.

### 2.2. Weighting

The weights prepared by the IQS Group were not calculated based on the cumulative distribution of the structural features. The IQS algorithm optimizes the found weights regarding the stability of the results, while minimizing the variance of weights and the range of weights, thus minimizing the mean square error of the test results. As a result, relatively low maximum weights and minimal mismatch of the final structure (after weighting) with the structure from the reference study were obtained; the maximum deviation for the category of the weighted variable was 3% of the share of a given category in the population. Table 1 presents detailed characteristics of the total sample (*N* = 1000).

The study group consisted of 885 people (those who were already vaccinated were excluded from the analysis) with a mean age of 47.43 with a median of 46 and a standard deviation of 16.02. Overall, 52.4% (464) was female and 47.6% (412) was male. 

**Figure 1:**
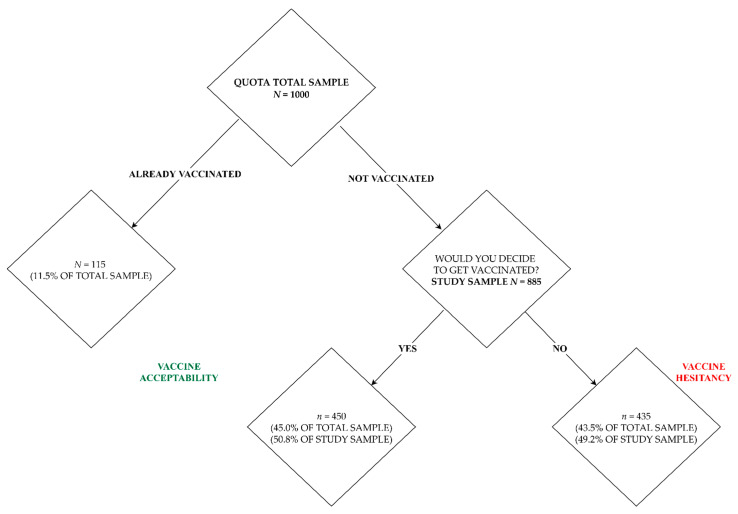
Study sample selection.

Table 2 presents additional characteristics of the study group. Figure 1 explains the study sample selection process. Tables presenting the distribution of the variables used in the regression models were included in the Supplementary Materials.

Due to the interdisciplinary nature of the project, the variables used for the study concerned health and psychosocial aspects and were based on other international projects [13,14]. In order to explain vaccine hesitancy in Poland, a sequential approach was used, where successive blocks (trajectories) of variables were introduced into the logistic regression analysis step by step (Table 3 and Table 4). The trajectories and the selection of variables were created after an in-depth discussion with the interdisciplinary project team, to express multidimensional background of an intention to be vaccinated. In every model, the interactions between variables were also analyzed when insignificant, not presented in the results (due to a large number of independent variables). The colors of trajectories presented in Table 3 were used and standardized for all results. Tables with descriptive statistics of variables used in regression models were attached as a Supplementary Materials (Tables S1–S35 in Excel file).

The baseline model consisted of general structural (demographic/anthropometric) variables: gender, age, level of own education and the best educated parent, household composition and marital status. Moreover, those variables were frequently reported as vaccination predictors in many studies [11,15,16,17,18,19,20,21].

An input of economic factors (occupational, marital status, assessment of the economic situation of the household at the time of the pandemic, difficulties in paying bills, debt and corresponding emotions) were included in the second model.

The third model introduced variables regarding beliefs about the pandemic and the evaluation of the institution’s actions. Respondents were asked about their attitudes to conspiracy beliefs according to the European Social Survey [13,14,22,23] (all index variables described in the Appendix A) of SARS-CoV-2 and its preventive methods, as well as and assessment of government actions in the context of dealing with the COVID-19 pandemic.

The fourth model involved health status variables: the history of coronavirus infection and the number of symptoms. Respondents who did not suffer from COVID-19 also declaratively assessed their health and physical fitness during the pandemic. Coronavirus convalescents did the same, but the reference was the disease, where questions were asked about their health and fitness after suffering from COVID-19. On this basis, a Fitness index was created (details in the Appendix A). The assumption of the index was to check the impact of the overall health condition of the respondent during a pandemic, measured by health and fitness, on the propensity to vaccinate. Are people highly validating their own physical fitness and health more saline for vaccination or vice versa?

We also included information on the panelist’s last stationary visit to a doctor during the last 12 months into the model, that is to say since the outbreak of the pandemic.

The fifth model involved variables and two indices related to the measurement of loneliness and isolation during the pandemic. For that purpose, we computed both the Loneliness and Social Distance index (details described in the Appendix A). 

The final model (full model) was to introduce psychosocial variables relating to faith and trust. We studied the declarative religiosity during the pandemic and the level of stress caused by daily reports on the pandemic. The respondents also expressed the level of trust in the government and scientists.

The data was analyzed using R (The R Project for Statistical Computing) with additional packages and IBM SPSS 26. Feature importance was calculated based on the state-of-the-art debiased Mean Decrease Impurity feature importance measure using out-of-bag sample feature selection from Random Forests (MDI-oob measure) [24]. Accuracy, sensitivity and specificity were calculated using a confusion matrix [25]. There were no missing data in this study due to the design of the survey, which obliged respondents to provide an answer. Nevertheless, there were categories of “no answer” to some questions, which were included in building the model (e.g., in socio-economic trajectories).

## 3. Results

We estimated the odds of vaccination using binary logistic regression, where the dependent variable included respondent answers to the question: “If the COVID-19 vaccine were offered to you today, would you decide to get vaccinated?” The distribution of attitudes was: 435 (49.2%) negative answers and 450 (50.8%) positive. 

**Figure 2:**
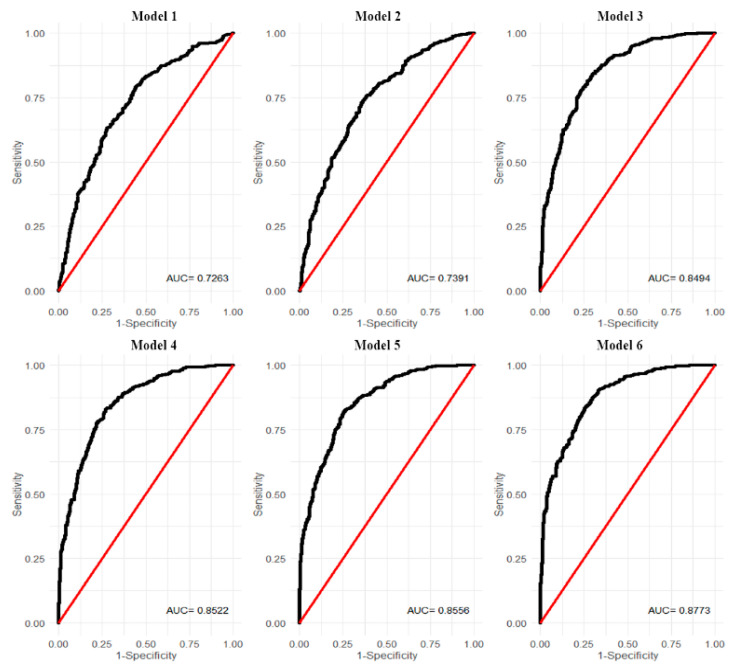
ROC curves for all models with Area Under the Curve (AUC).

The first (demographic) model was the baseline for further stepwise analysis. Several factors stand out as significant predictors of increased odds of vaccination (Table 5): age (OR = 1.05, CI = (1.04–1.06), male gender (OR = 1.51, CI = (1.13–2.03)), living in cities with more than 100,000 inhabitants (OR = 1.64, CI = (1.14–2.36)), and secondary or post-secondary education in comparison to basic vocational or lower education (OR = 1.58, CI = (1.12–2.22)). Interestingly, having a parent with a bachelor’s degree or higher also increases the odds of being vaccinated by 86% compared to respondents whose parents had a basic vocational or lower education (OR = 1.86, CI = (1.14–3.04)). Accuracy, sensitivity, and specificity for this model were 0.67, 0.66, and 0.67 respectively. To ensure that the validity of the calibration was assessed, the ROC curve was plotted and the Area Under ROC Curve (AUC) was calculated. In this model, the AUC is 0.7263 (Figure 2). The VIF (Independent Variable Collinear Test) for each variable used in the model did not exceed 1.5. 

In addition to the demographic trajectory, the second model included economic variables. However, only one of the economic predictors was significant: difficulty in paying bills in the last 12 months. The odds of being vaccinated among people who refused to answer the question were higher by 219% in comparison to people who declared to have no problems with paying their bills (OR = 3.19, CI = (1.43–7.29)).

In the third model, variables regarding beliefs about the pandemic were added to the two previous trajectories. Demographic and economic variables that were significant in the earlier models remained significant. Additionally, the economic variable household debt, i.e., 98—“don’t know”, was found to be significant and the odds of vaccination decreases by 44% with respect to the reference category of this variable: 1—indebted (OR = 0.56, CI = (0.31–0.99). Those who could not or would not identify their debt showed lowere willingness to vaccinate in comparison to those who admitted to household debt. From the group of variables regarding beliefs about the pandemic, three had a significant effect on the odds: Conspiracy Index (OR = 0.80, CI = (0.73–0.88)), attitude towards the coronavirus (OR = 1.32, CI = (1.20–1.45)) and tendency to wear a mask (OR = 1.47, CI = (1.27–1.71)).

A fourth model, with the group of variables related to health status, was subsequently built. There was only one significant factor—Fitness Index (OR = 0.84, CI = (0.72–0.98)). Moreover, personal or household COVID-19 history and seeing a medical doctor during the last 12 months had no significant impact on vaccination decision.

The fifth step was putting the next trajectory into the model (social isolation factors). The odds of being vaccinated was significant and increased for the variable Social Distance Index, as this variable increases by a unit of 4% (OR = 1.04, CI = (1.01–1.08)). Simultaneously, the Loneliness index was insignificant. 

**Figure 3:**
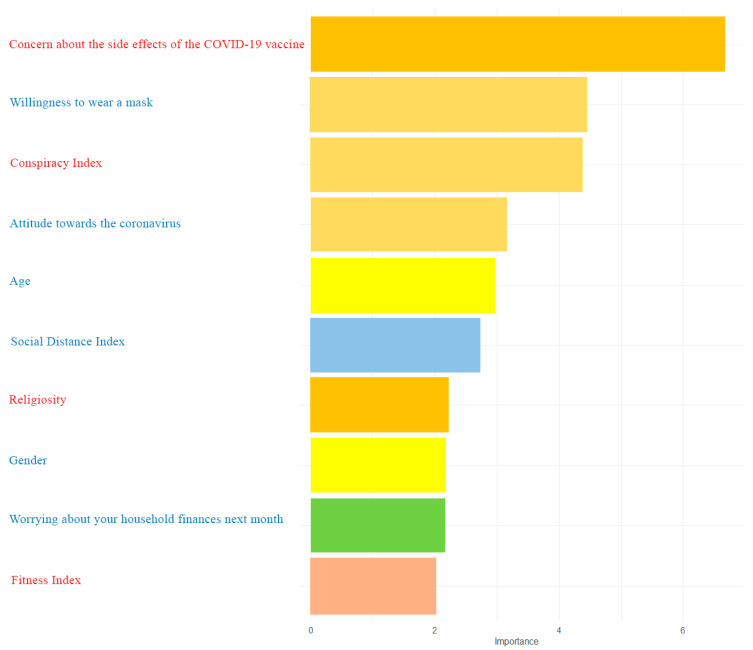
Random forest variable importance. Blue variable cause an increase in vaccine acceptability, while red variables cause a decrease.

For the final model, psychosocial variables related to belief and trust were introduced. People worrying about vaccine side effects were 82% less likely to be vaccinated (OR = 0.18, CI = (0.11–0.30). In the final model, we detected an impact of variables that were also significant in previous models, these are strong variables that increase or decrease the odds. However, several variables had no effect when more trajectories were taken into account (Table 6). In Figure 3, we also presented the importance of particular variables for the final model (including colors of trajectories). The VIF (test of collinearity of independent variables) for each variable used in the final model does not exceed a value of 4. Final model accuracy is 0.78, sensitivity is 0.78, and specificity is 0.79, AUC = 0.877 for the ROC curve. Considering all our proposed trajectories, a greater amount of data allows for a more detailed explanation of the phenomenon of vaccine hesitancy/acceptability. Moreover, analyzing a limited number of variables may contain a potential inference bias.

Random forests were then used to rank the importance of variables in a logistic regression (final) model. Figure 3 shows the variable importance for the 10 most significant variables used in the model.

## 4. Discussion

Research on vaccine hesitancy or acceptability can be divided into two groups concerning the level of the phenomenon in the analyzed populations and the factors determining this level. The first of these goals is usually based on respondents’ declarations, while the second is much more difficult, including multivariate analysis with their intensity and interactions. In the population we studied, the distribution of supporters and skeptics of vaccination against COVID-19 was almost even, which was favorable to the attempt at explanatory modeling.

The level of vaccine hesitancy in many published studies varied depending on whether the studies covered the general population or only a subpopulation separated on the basis of certain characteristics (e.g., health care workers, administration or students). The acceptance of vaccination against COVID-19 was in many studies associated with epidemiological factors such as mortality and morbidity [11,26]. The highest vaccine acceptance rates in the general population have so far been found in Ecuador (97.0%) [27], Indonesia (93.3%) [28], Malaysia (94.3%) [15] and China (91.3%) [29]. On the other hand, several studies showed significantly lower percentages of positive attitudes towards vaccination: Kuwait 23.6% and Jordan 28.4% [10], Italy 53.7% [30], Russia 54.9% and France 58.9% [11]. It should be noted that, especially in the case of European countries, the number of studies on vaccine hesitancy against COVID-19 is high, and oftentimes, the results of studies in the general population provided a different picture of society. For example, studies in the UK indicated the level of acceptance for vaccination as 64.0% [31], 71.7% [32], up to 79.0% [33] or 83.0% [34]. Similarly, higher than the aforementioned acceptability levels were obtained in other studies in France [35] and Italy [11]. These discrepancies could be explained by the month in which the research was carried out; several studies have found that after the end of waves, with increased morbidity or because of disinformation in communication about vaccine effectiveness, the percentage of people willing to get vaccinated decreased [36,37,38].

Thus far, a few studies have assessed the willingness to vaccinate against COVID-19 in the general population of Poland. In the June 2020 study, it was found that 56.3% (*N* = 666, age >= 18) showed a positive attitude towards vaccination [11], while in a parallel (June 2020) study with the quota sampling method (*N* = 1066), 37.0% declared vaccination susceptibility [16]. Interesting results, covering a much larger, representative group of the studied group (~6000 divided into two waves), were obtained in an experiment consisting of presenting persuasive messages related to vaccination (e.g., effectiveness and social popularity) before asking the question about the attitude towards vaccinations [17]. The research was carried out in 2021—after the first wave in January and the second in March. After the first series of tests, when asked about attitudes towards vaccination, the results were clearly similar to ours: 25.9% “definitely yes”, 26.6% “rather yes”, 26.5% “rather not”, and 21.6% “definitely not”. In the second wave, the situation changed slightly: 24.1% “definitely yes”, 30.8% “rather yes”, 24.6% “rather not”, and 20.5% “definitely not”. In particular, this study is consistent with the results presented by us, and in conjunction with other studies, it suggests that in Poland, regardless of changes in the intensity of the pandemic, the percentage of people who are skeptical about vaccination remains high.

In addition to establishing the acceptance level for vaccination in the general population, the aim of our study was to identify factors that determine vaccine hesitancy/acceptability. Some of the factors expressed in the trajectories we proposed were investigated by researchers dealing with vaccine hesitancy. The most frequently analyzed determinants were demographic factors. As in our study, several studies indicated a negative relationship between the acceptability of vaccinations and age [11,18,19,20], but other polls found high percentages of positive attitudes towards vaccination in age groups between 18 and 34 years of age [21]. Studies on the Polish population have so far shown that the tendency to vaccinate increases linearly with age [17]; on the other hand, people between 25 and 44 years of age are significantly less likely to be vaccinated against COVID-19 [16]. In our study, we showed OR = 1.05 for the age predictor. Similar to our findings, most studies indicated a lower willingness to vaccinate among women [10,18,19,39], and just a few studies suggest the opposite [11,15,35].

Among other factors related to vaccine hesitancy, one can distinguish those that focus on the attributes of the vaccines themselves or the effects of its administration, such as safety [40], side effects [18,33], efficacy [41,42] and the short or unknown duration of resistance they offer [40,41,42]. The second group of variables are the general attitudes towards vaccination related to anti-vaccine movements [43], and insufficient information from vaccine manufacturers [44]. A very important predictor of abandonment of vaccination was believing in conspiracy theories [45], which was reflected in a previous Polish study [17] as well as ours. The list of factors increasing the motivation to vaccinate also includes recommendations of medical personnel [40,41,46] or opinions expressed by relatives [41].

## 5. Conclusions

In the cited Polish studies, among the factors that significantly determined the positive will to be vaccinated were the presentation of scientific research on vaccine safety, the inability to travel without vaccination and the possibility of introducing financial penalties for the unvaccinated [16], living in large cities of over 500,000 inhabitants (in our study over 100 thousand), having higher education, relatives passing away from COVID-19, wealth, and even left-wing political views [17,43]. It should be noted, however, that one of the Polish studies [17] was partially interventional (an interview induced by information presented to the respondent on vaccination and COVID-19). Thus far, apart from the studies discussed above, most of the Polish studies on COVID-19 vaccine hesitancy have focused on selected professional [47,48] and societal groups [49,50]. Our study addresses the problem of vaccine hesitancy in the broadest way through multivariate statistical modeling, and primarily refers to the general population.

Numerous studies have addressed the problem of complacency about SARS-CoV-2 [51] and the presence of chronic diseases determining the increased willingness to vaccinate [52,53]. In our study, the Fitness Index shows a similar association.

Vaccine hesitancy is a problem that has been known for years, but it takes on a special dimension during the COVID-19 pandemic. Based on the literature, particular attention should be paid to those populations most affected by skepticism. Central and Eastern European countries, including Poland, represent such populations. Our goal is to provide additional knowledge on factors regulating the willingness to vaccinate—other than typical ones found in previous research (gender, age, education and belief in conspiracy theories). Our study paints a larger picture, in which we attempted to account for the interdisciplinary context of influencing the decision to vaccinate. It must also be emphasized that analyzing vaccine hesitancy or acceptability from the perspective of a narrow group of variables may create the risk of inference bias. Therefore, it is necessary to correct the models or supplement them with additional groups of variables. Our study attempts to provide a broader answer by revealing some of the psychosocial characteristics behind an unwillingness to vaccinate. The stepwise process of introducing variables into the model allowed us to show which variables maintain significance and how strongly they explain the reason or reasons for the reluctance to vaccinate against SARS-COV-2. On this basis, it is possible to identify several psychosocial characteristics of people adverse to vaccination. Currently, reluctant individuals are primarily those who ‘fear the side effects of the COVID-19 vaccine’. In addition, they are also people unaffected by the coronavirus and very healthy and physically fit (Fitness index). Then there are people with lower levels of education, from smaller towns, struggling financially and who have trouble paying their bills in the last 12 months and ‘worry about their household finances next month’; they also tend to be conservative, as measured by the degree of religiosity and the number of people in a household. Another factor is being unconvinced about the scale and degree of risk of the COVID-19 pandemic. This is confirmed by the Conspiracy Index, the tendency to wear a mask and the Social Distance Index.

Our aim was to support health policy makers in designing education-based public health interventions. Understandably, not all factors determining vaccine hesitancy can be addressed using this strategy, e.g., economic factors such as concerns about household finances, which were clearly demonstrated in our study. There are two crucial concepts that can help change the direction of vaccination perception: knowledge and a recommendation system (communication). Knowledge, i.e., the result of research focused on the questionable issues (in public perception) should be shared with the general population in a comprehensive and personalized manner (according to the educational attainment). The recommendation system should focus on health care professionals in accordance with the principles of Evidence Based Medicine. Communication chaos creates a sense of insecurity and undermines authorities (government and science). Effective campaigns promoting vaccination should be considered on the decentralized, local level; WHO leadership would be advantageous.

## Figures and Tables

**Table 1:** Characteristics of the total sample (*N* = 1000).

Variables		Local Data Bank Sample Structure *N* = 1000	Obtained Sample *N* = 1000	Weighted Sample *N* = 1000
Gender and age	woman 18–24	4.5%	4.0%	3.9%
	woman 25–34	8.8%	9.3%	9.3%
	woman 35–44	9.7%	9.6%	9.5%
	woman 45–54	7.6%	7.7%	7.7%
	woman 55–64	8.8%	8.6%	8.3%
	woman 65+	12.9%	13.3%	13.5%
	man 18–24	4.7%	4.2%	4.5%
	man 25–34	9.1%	8.9%	8.9%
	man 35–44	9.9%	10.2%	10.1%
	man 45–54	7.6%	7.5%	7.6%
	man 55–64	8.0%	8.1%	8.0%
	man 65+	8.5%	8.6%	8.6%
Place of residence	rural	39.2%	38.5%	39.0%
	city up to 15,000 residents	10.1%	10.0%	10.0%
	city 15–99 thousand residents	21.3%	22.5%	22.4%
	city 100–999 thousand residents	22.0%	22.6%	22.3%
	city with over 1,000,000 residents	6.3%	6.4%	6.3%
Education	lower	40.8%	39.5%	40.4%
	medium	34.8%	36.0%	35.1%
	higher	24.5%	24.5%	24.5%
Voivodship	Lower Silesia	7.7%	7.6%	7.6%
	Kuyavian-Pomeranian	5.4%	5.7%	5.5%
	Lublin	6.5%	6.5%	6.5%
	Lubusz	5.5%	5.8%	5.6%
	Lodzkie	2.6%	2.9%	2.7%
	Lesser Poland	8.8%	8.6%	8.7%
	Masovia	13.9%	13.3%	13.8%
	Opole	2.6%	2.8%	2.7%
	Subcarpathia	5.5%	5.8%	5.6%
	Podlasie	3.1%	3.2%	3.1%
	Pomerania	6.0%	5.7%	5.9%
	Silesia	12.0%	10.9%	11.8%
	Świętokrzyskie Province	3.3%	3.3%	3.3%
	Warmia-Masuria Province	3.7%	3.8%	3.7%
	Greater Poland	9.0%	9.2%	9.0%
	West Pomerania	4.5%	4.9%	4.5%

**Table 2:** Characteristics of the study sample.

Variables		*N* = 885	
		*n*	%N
Gender	Female	464	52.4%
	Male	421	47.6%
Education	basic vocational or lower	368	41.5%
	secondary or post-secondary	326	36.9%
	Bachelor’s degree or higher	191	21.6%
Place of residence	>100 k inhabitants	244	27.5%
	<100 k	289	32.7%
	rural	352	39.8%
Age	Mean	47.43	
	Median	46.00	
	SD	16.028	
	Min	19	
	Max	80	

**Table 3:** Trajectories and variables use for the logistic regression models.

	Heading	Variables Used for Trajectories
**Impact Trajectories**	Demographic	Age
		Place of residence
		Gender
		Education
		Parents education
	Socio-economic	Number of people in the household
		Marital status
		Difficulty in paying bills in the last 12 months
		Time during which expenses can be covered by savings
		Worrying about household finances in next month
		Household debt
		Occupational status
	Attitudes	Conspiracy Index
		The legitimacy of government restrictions
		Acceptance of government restrictions
		Attitude towards the coronavirus
		Assessment of restrictions applied by the government
		Willingness to wear a mask
	Health-related	SARS-CoV-2 history
		SARS-CoV-2 history in the household
		COVID-19 symptoms
		Fitness Index
		Visit to the doctor in the last 12 months
	Social-isolation	Loneliness Index
		Sense of love and trust
		Social Distance Index
	Faith and trust	Religiosity
		Stress experiencing due to pandemic
		Trust in government
		Trust in scientists Concern about the side effects of the COVID-19 vaccine

**Table 4:** Stepwise algorithm of trajectories in the models.

Trajectories					
Demographic	Socio-Economic	Attitudes	Health-Related	Social-Isolation	Faith and Trust
Model 1					
Model 2					
Model 3					
Model 4					
Model 5					
Model 6					

**Table 5:** Demographic predictors of vaccine acceptability (‘Would you get vaccinated against COVID-19 (y/n)?’).

Heading	Vaccination Acceptability (Yes)
Predictors	Odds Ratios and 95%CI
(Intercept)	0.06 *** (0.03–0.10)
Age	1.05 *** (1.04–1.06)
Place of residence: 1—above 100 thous. ref = “3—rural”	1.64 ** (1.14–2.36)
Place of residence: 2—less than 100 thous. ref = “3—rural”	1.20 (0.85–1.69)
Gender: 2—men ref = “1—woman”	1.51 ** (1.13–2.03)
Education: 2—secondary or post-secondary ref = “1—basic vocational or lower”	1.58 ** (1.12–2.22)
Education: 3—bachelor’s degree or higher ref = “1—basic vocational or lower”	1.42 (0.92–2.20)
Parents education: 2—secondary or post-secondary ref = “1—basic vocational or lower”	1.26 (0.90–1.77)
Parents education: 3—bachelor’s degree or higher ref = “1—basic vocational or lower”	1.86 * (1.14–3.04)
Parents education: 99—no answer ref = “1—basic vocational or lower”	1.00 (0.50–1.98)
Observations	885
R^2^ Tjur	0.150
AIC	1107.142
log-Likelihood	−543.571

Note: * *p* < 0.05, ** *p* < 0.01, *** *p* < 0.001.

**Table 6:** Logistic regression model coefficients.

	Vaccination Acceptability/Hesitancy				
Predictors	Model 2 OR (95 CI)	Model 3 OR (95 CI)	Model 4 OR (95 CI)	Model 5 OR (95 CI)	Model 6 OR (95 CI)
(Intercept)	0.06 *** (0.02–0.15)	0.01 *** (0.00–0.05)	0.03 *** (0.01–0.15)	0.02 *** (0.00–0.09)	0.07 ** (0.01–0.47)
Age	1.04 *** (1.03–1.06)	1.03 *** (1.01–1.04)	1.02 ** (1.01–1.04)	1.02 ** (1.01–1.04)	1.02 ** (1.01–1.04)
Place of residence: 1—above 100 thous. ref = 3-rural	1.58 * (1.08–2.30)	1.56 * (1.02–2.41)	1.56 * (1.01–2.41)	1.56 * (1.01–2.43)	1.51 (0.96–2.41)
Place of residence: 2—less than 100 thous. ref = 3—rural	1.14 (0.80–1.63)	1.36 (0.90–2.05)	1.37 (0.91–2.08)	1.36 (0.90–2.07)	1.38 (0.89–2.13)
Gender: 2—man ref = 1—women	1.51 * (1.10–2.07)	1.54 * (1.07–2.21)	1.49 * (1.04–2.15)	1.67 ** (1.15–2.45)	1.56 * (1.05–2.32)
Education: 2—secondary or post-secondary ref = 1—basic vocational or lower	1.49 * (1.05–2.11)	1.46 (0.97–2.20)	1.41 (0.93–2.13)	1.35 (0.89–2.07)	1.24 (0.79–1.92)
Education: 3—bachelor’s degree or higher ref = 1—basic vocational or lower	1.35 (0.85–2.13)	1.16 (0.68–1.97)	1.07 (0.62–1.83)	1.05 (0.61–1.81)	1.02 (0.57–1.81)
Parents education: 2—secondary or post-secondary ref = 1—basic vocational or lower	1.35 (0.96–1.91)	1.40 (0.93–2.10)	1.39 (0.92–2.08)	1.43 (0.95–2.15)	1.29 (0.84–1.99)
Parents education: 3—bachelor’s degree or higher ref = 1—basic vocational or lower	1.80 * (1.10–2.97)	1.64 (0.93–2.92)	1.66 (0.94–2.96)	1.64 (0.92–2.95)	1.45 (0.79–2.68)
Parents education: 99—no answer ref = 1—basic vocational or lower	0.98 (0.48–1.97)	1.19 (0.52–2.70)	1.28 (0.56–2.91)	1.23 (0.53–2.82)	1.16 (0.48–2.77)
Number of people in the household	0.94 (0.85–0.99)	0.96 (0.86–1.00)	0.96 (0.86–1.00)	0.96 (0.86–1.00)	0.96 (0.86–1.00)
Marital status: 2—being in a relationship ref = 1—single	1.01 (0.74–1.39)	1.00 (0.69–1.43)	1.04 (0.72–1.51)	1.06 (0.73–1.55)	1.15 (0.77–1.72)
Difficulty in paying bills in the last 12 months: 1—problems with paying bills in the last 12 months ref = 2—no problems with paying bills in the last 12 months	1.34 (0.92–1.97)	1.17 (0.76–1.82)	1.13 (0.73–1.76)	1.13 (0.72–1.77)	1.08 (0.67–1.72)
Difficulty in paying bills in the last 12 months: 99—no answer ref = 2—no problems with paying bills in the last 12 months	3.19 ** (1.43–7.29)	3.42 ** (1.42–8.52)	3.00 * (1.23–7.52)	3.12 * (1.27–7.93)	2.50 (0.97–6.56)
Period of possibility to pay for current expenses from savings: 2—from 1 to 3 months ref = 1—less than 1 month	1.26 (0.83–1.90)	1.20 (0.74–1.93)	1.20 (0.74–1.95)	1.17 (0.72–1.91)	1.09 (0.66–1.81)
Period of possibility to pay for current expenses from savings: 3—over 3 months ref = 1—less than 1 month	1.18 (0.74–1.87)	1.03 (0.60–1.77)	1.02 (0.59–1.76)	1.00 (0.57–1.73)	0.86 (0.48–1.52)
Period of possibility to pay for current expenses from savings: 98—don’t know ref = 1—less than 1 month	0.93 (0.57–1.53)	0.73 (0.41–1.30)	0.77 (0.43–1.38)	0.78 (0.43–1.40)	0.81 (0.44–1.51)
Worrying about your household finances next month: 2—not worried ref = 1—worried	1.39 (0.99–1.95)	1.47 (0.99–2.18)	1.54 * (1.04–2.31)	1.56 * (1.05–2.35)	1.61 * (1.05–2.49)
Household debt: 2—not indebted ref = 1—indebted	0.95 (0.68–1.34)	0.95 (0.64–1.41)	0.94 (0.63–1.41)	0.95 (0.63–1.42)	0.84 (0.55–1.28)
Household debt: 98—don’t know ref = 1—indebted	0.72 (0.44–1.19)	0.56 * (0.31–0.99)	0.54 * (0.30–0.97)	0.54 * (0.30–0.97)	0.54 (0.29–1.02)
Occupational status: 2—unemployed ref = 1—employed	1.05 (0.75–1.46)	0.84 (0.57–1.24)	0.82 (0.55–1.22)	0.79 (0.53–1.18)	0.77 (0.50–1.16)
Occupational status: 99—no answer ref = 1—employed	1.96 (0.46–8.23)	3.03 (0.64–14.24)	3.62 (0.73–17.48)	3.65 (0.71–18.37)	4.48 (0.80–25.77)
Conspiracy Index 0—no belief in conspiracy theories; 10—strong belief in conspiracy theories		0.80 *** (0.73–0.88)	0.80 *** (0.73–0.88)	0.79 *** (0.72–0.87)	0.80 *** (0.73–0.88)
The legitimacy of government restrictions 0—completely illegitimate; 10—completely legitimate		0.99 (0.90–1.09)	1.00 (0.90–1.10)	0.99 (0.90–1.09)	1.05 (0.94–1.16)
Acceptance of government restrictions 0—not at all acceptable; 10—fully acceptable		0.97 (0.88–1.08)	0.97 (0.88–1.08)	0.97 (0.87–1.08)	0.95 (0.85–1.06)
Attitude towards the coronavirus 0—it does not pose a threat to the health and life of citizens at all; 10—poses a very serious threat to the health and life of citizens		1.32 *** (1.20–1.45)	1.30 *** (1.18–1.43)	1.24 *** (1.12–1.37)	1.19 ** (1.07–1.33)
Assessment of restrictions applied by the government 0—Too small for the scale of the threat posed by the COVID-19 pandemic to the life and health of citizens; 10—Too large for the scale of the threat posed by the COVID-19 pandemic to the life and health of citizens		1.01 (0.94–1.09)	1.02 (0.95–1.10)	1.02 (0.95–1.10)	1.02 (0.95–1.11)
Tendency to wear a mask 1—definitely not; 5—definitely yes		1.47 *** (1.27–1.71)	1.49 *** (1.29–1.74)	1.42 *** (1.21–1.67)	1.46 *** (1.24–1.72)
SARS-CoV-2 personal history: 1—yes; 2—no			1.16 (0.67–2.02)	1.15 (0.66–2.00)	1.15 (0.64–2.06)
SARS-CoV-2 in the household: 1—yes; 2—no			0.96 (0.55–1.68)	0.99 (0.56–1.74)	0.97 (0.54–1.72)
COVID-19 symptoms: 1—1–4 symptoms out of 22 ref = 0—no symptoms			1.02 (0.60–1.74)	0.99 (0.58–1.69)	0.91 (0.52–1.61)
COVID-19 symptoms: 2—5 or more symptoms out of 22 ref = 0—no symptoms			0.93 (0.54–1.60)	0.91 (0.53–1.57)	0.83 (0.47–1.46)
Fitness Index			0.84 * (0.72–0.98)	0.86 (0.74–1.02)	0.84 * (0.70–1.00)
Visit to the doctor in the last 12 months			1.00 (0.99–1.00)	1.00 (0.99–1.00)	0.99 (0.99–1.00)
Loneliness Index				1.09 (0.97–1.23)	1.12 (0.99–1.28)
Sense of love and trust: 2—no ref = 1—yes				0.85 (0.49–1.46)	0.76 (0.43–1.34)
Sense of love and trust: 99—no answer ref = 1—yes				1.12 (0.54–2.29)	1.16 (0.54–2.48)
Social Distance Index				1.04 * (1.01–1.08)	1.05 ** (1.01–1.09)
Degree of religiosity: 0—not religious at all; 10—very religious					0.93 * (0.87–0.99)
Everyday stress caused by a pandemic: 0—not stressful at all; 10—causes constant stress					1.06 (0.98–1.15)
Trust in government: 0—no trust at all; 10—complete trust					0.99 (0.96–1.01)
Trust in scientists: 0—no trust at all; 10—complete trust					1.00 (0.98–1.02)
Concern about the side effects of the COVID-19 vaccine: 2—concerned ref= 1—not concerned					0.18 *** (0.11–0.30)
Observations	885	885	885	885	885
R^2^ Tjur	0.172	0.369	0.376	0.381	0.432
AIC (Akaike’s Information Criterion)	1107.444	905.000	909.892	908.504	855.322
log-Likelihood	−531.722	−424.500	−420.946	−416.252	−384.661

Note: * *p* < 0.05, ** *p* < 0.01, *** *p* < 0.001.

## Data Availability

The dataset we generated and/or analyzed during the current study are not publicly available due to confidentiality issues but are available from the corresponding author on request.

## Supplementary Materials

Tables with descriptive statistics of variables used in regression models are available online https://www.mdpi.com/article/10.3390/vaccines9080876/s1.

## Appendix A

We supplement hereby a methodological description of variables that were recoded first and then used to create indices, i.e., Conspiracy, Fitness, Loneliness and Social Distance.

The conspiracy index: Panelists were asked about their attitudes to conspiracy beliefs, according to the European Social Survey. They were presented with statements for evaluation on a 5-point Likert scale (1 ‘strongly disagree’, 2 ‘rather agree’, 3 ‘I neither agree nor disagree’, 4 ‘I rather agree’, 5 ‘I strongly agree’, 9 ‘don’t know’). Based on the statements, an index of conspiracy beliefs was created, where positive responses on the scale (4 and 5) were recoded as 1, while all other options were recoded as 0. Thus, summing up the responses from the six statements, the index took values from 0, ‘complete lack of believe in conspiracy theories’, to 6, ‘very strong belief in conspiracy theories’. Statements used for the index were as follows (http://www.europeansocialsurvey.org/docs/about/COVID-19-Conspiracy-Beliefs.pdf; http://www.europeansocialsurvey.org/docs/about/ESS010-COVID-19-module.pdf):

1. The coronavirus (COVID-19) was created in a laboratory as a biological weapon.
2. Certain significant events have been the result of the activity of a small group who secretly manipulate world events.
3. Groups of scientists manipulate, fabricate or suppress evidence in order to deceive the public.
4. Governments should let people make their own decisions about how to best protect themselves and their loved ones from the COVID-19 virus.
5. Coronavirus is the result of deliberate and concealed efforts of some government or organization.
6. Some vaccines have chips that allow people to be controlled.

The fitness index: Respondents who did not have COVID-19 declaratively assessed their health and physical fitness during the pandemic (5 ‘perfect’, 4 ‘very good’, 3 ‘good’, 2 ‘not so good’, 1 ‘bad’, 98 ‘don’t know’). Coronavirus convalescents did the same, but the reference was the disease, where questions were asked about their health and fitness after suffering COVID-19. On this basis, a fitness index was created with values from 2 to 6. Responses were recoded into the following form: 1 ‘bad and not so good’, 2 ‘good’, 3 ‘very good or perfect’, and were then summed for health and fitness.

The loneliness index: This index ranged from 0 to 4, was built on the basis of four variables assessed on a scale (4 ‘often’, 3 ‘sometimes’, 2 ‘rarely’, 1 ‘never’). The respondents answered how often: ‘Do you feel you are missing the company of others?’, ‘Do you feel sidelined?’, ‘Do you feel isolated from others?’ and ‘Do you feel lonely?’ The responses were then recoded into the form: 1 ‘often or sometimes’ and 0 ‘rarely or never’, and summed up to give the loneliness index.

The social distance index: This index was based on three questions: ‘How often in the last two months have you intentionally avoided contact with other people?’, ‘How often in the last two months have you kept the minimum distance 1.5 m during contacts outside the home?’, ‘Did you cover your mouth and nose or just your mouth in a public place?’. All responses were measured on a scale from 0—never to 10—always. The index was the sum of these values, ranging from 0 to 30.
